# Zn(II)-Coordinated Quantum Dot-FRET Nanosensors for the Detection of Protein Kinase Activity

**DOI:** 10.3390/s150817977

**Published:** 2015-07-23

**Authors:** Butaek Lim, Ji-In Park, Kyung Jin Lee, Jin-Won Lee, Tae-Wuk Kim, Young-Pil Kim

**Affiliations:** 1 Department of Life Science, Hanyang University, Seoul 133-791, Korea; E-Mails: plobere804@naver.com (B.L.); skshzzang@nate.com (J.-I.P.); jwl@hanyang.ac.kr (J.-W.L.); 2 Research Institute for Natural Sciences, Hanyang University, Seoul 133-791, Korea; 3 Department of Convergence Medicine, Asan Institute for Life Sciences, University of Ulsan College of Medicine, Asan Medical Center, Seoul 138-736, Korea; E-Mail: kjlee@amc.seoul.kr; 4 Research Institute for Convergence of Basic Sciences, Hanyang University, Seoul 133-791, Korea

**Keywords:** FRET, quantum dot, zinc, kinase, phosphorylation

## Abstract

We report a simple detection of protein kinase activity using Zn(II)-mediated fluorescent resonance energy transfer (FRET) between quantum dots (QDs) and dye-tethered peptides. With neither complex chemical ligands nor surface modification of QDs, Zn(II) was the only metal ion that enabled the phosphorylated peptides to be strongly attached on the carboxyl groups of the QD surface via metal coordination, thus leading to a significant FRET efficiency. As a result, protein kinase activity in intermixed solution was efficiently detected by QD-FRET via Zn(II) coordination, especially when the peptide substrate was combined with affinity-based purification. We also found that mono- and di-phosphorylation in the peptide substrate could be discriminated by the Zn(II)-mediated QD-FRET. Our approach is expected to find applications for studying physiological function and signal transduction with respect to protein kinase activity.

## 1. Introduction

Protein kinases have been recognized as one of the largest families of cell-regulatory molecules with more than 500 encoded in the human genome [1,2]. Their catalytic activities, therefore, need to be characterized to understand the transmission of signals in a myriad of biological events. Traditional kinase assays have primarily relied upon radioisotopic labeling using gamma-^32^P-adenosine triphosphate (ATP) [3,4] or non-radioisotopic immunoblotting using anti-phosphopeptide antibodies [5,6,7], but these are labor-intensive processes. Fluorescent resonance energy transfer (FRET)-based detection of protein kinase activity has emerged as an alternative [8,9], due to its simplicity, ratiometric accuracy, and time-lapse sensitivity. To monitor protein kinase activity in living cells, FRET-based reporters have been developed by using genetically encoded fluorescent proteins linked to phosphoaminoacid-binding domain and kinase substrate sequence. Over the last few decades, many attempts based on FRET sensors have been made in a rapid format for *in vitro* drug screening [10,11,12,13]. However, there is still a challenge in enhancing FRET efficiency to avoid high background noise signal. To resolve this problem, it is important to note that quantum dot (QD)-based FRETs are capable of generating higher energy transfer efficiency with greater sensitivity compared to general FRET couplers [14,15,16,17,18], owing to their improved optical properties, including high extinction cross-section, high quantum yield, and long photoluminescence lifetime. However, much less effort has been made to design QD-coupled FRET for the detection of protein kinase activity. Although a few applications of QD-FRET sensors to assay peptide phosphorylation have been reported [19,20,21], the use of either positively-charged peptides dependent on environmentally-labile interactions or phosphate-specific antibodies with large sizes still impedes the achievement of a simple QD-FRET design. To design a facile sensor, it is desirable for a new type of QD-FRET system to induce the FRET change by peptide phosphorylation with high affinity under antibody-free conditions.

Here we propose a simple QD-FRET method for detecting phosphorylated peptides via Zn(II) coordination. Since Zn(II) naturally has a strong interaction with phosphate ions in zinc-binding enzymes [22,23], and is also well-coordinated with multidentate ligands based on metal binding affinity [24], we reason that phosphopeptides may be preferably associated via Zn(II) coordination with the functional groups (*i.e*., carboxyl groups) of a QD surface that may act as multidentate ligands. This improved association can be realized, based on the increased surface density of functional groups due to the large surface-to-volume ratio of the nanoparticles. We recently reported Zn(II)-mediated self-assembly of gold nanoparticles with phosphopeptides [25], but this system was useful for peptide dephosphorylation rather than phosphorylation, due to reduced detection sensitivity by excess ATP during protein kinase reaction. Therefore, we envisage that QD-FRET could be an alternative to avoid this issue.

## 2. Experimental Section

### 2.1. Materials

Zinc(II) chloride (98%, ZnCl_2_), nickel(II) chloride hexahydrate (99.9%, NiCl_2_·6(H_2_O)), cobalt(II) chloride hexahydrate (CoCl_2_·6H_2_O), iron(III) chloride hexahydrate (98%, FeCl_3_·6H_2_O), magnesium chloride hexahydrate (MgCl_2_·6H_2_O), adenosine 5′-triphosphate (ATP) disodium salt hydrate (99%, 5′-ATP-Na_2_), α-cyano-4-hydroxycinnamic acid (98%, CHCA) and trifluoroacetic acid (99%, TFA) were purchased from Sigma-Aldrich (St. Louis, MO, USA). Copper(II) chloride dihydrate (CuCl_2_·2H_2_O) was purchased from Junsei Chemical Co., Ltd. (Tokyo, Japan). Carboxyl quantum dots (Qdot525 ITK carboxyl) and streptavidin agarose were purchased from Life Technologies Inc., Carlsbad, CA, USA. The catalytic subunit of protein kinase A (PKA) was purchased from New England Biolabs (NEB). Tetramethyl-6-carboxyrhodamine (TAMRA)-labeled peptides (T-PEP1, TAMRA-LRRASLG; T-pPEP1, TAMRA-LRRApSLG; T-PEP1-Bio, TAMRA-LRRASLGK-Biotin; T-PEP2, TAMRA- IYAAPKKG; T-pPEP2, TAMRA-IpYAAPKKG; T-PEP3, TAMRA-KEEPPSPPQSPR; T-pPEP3, TAMRA-KEEPPSPPQpSPR; T-ppPEP3, TAMRA-KEEPPpSPPQpSPR) were synthesized by Peptron Inc., Daejeon, Korea. All other chemicals were of analytical grade and were used as received.

### 2.2. FRET Measurement

A TAMRA-labeled peptide substrate (8 µL at 1 µM) and carboxyl QD525 (20 µL at 10 nM) were mixed in 20 mM Tris-HCl buffer (pH 7.4) to give a final volume of 99 µL, then metal ion stock (1 µL at 10 mM) in 20 mM Tris-HCl buffer (divalent or trivalent metal ions) was added to the mixture. This mixture was incubated for 5 min at RT with gentle mixing. The final concentrations of the QD and peptide in the aqueous solution were 2 nM and 80 nM, respectively. The concentrations of the QD and TAMRA-peptide were determined using the respective extinction coefficients 130,000 M^−1^·cm^−1^ at 488 nm and 65,000 M^−1^·cm^−1^ at 555 nm. Fluorescence spectra were obtained from 450 nm to 650 nm (at an excitation wavelength of 380 nm) using a spectrofluorometer (FS-2, Scinco Inc., Seoul, Korea). The fluorescent emission of TAMRA at an excitation of 380 nm was minimized in the absence of QDs. The FRET ratio was determined by dividing the acceptor integrated emission (550–650 nm) by the donor integration emission (450–550 nm). To determine the binding affinity of Zn(II) ion in relation to QD-FRET signal, FRET signal was measured as a function of Zn(II) ion. The highest FRET ratio was converted into 100% which was set to maximum. The binding affinity expressed as a dissociation constant (*K*_d_) was calculated from half maximal concentration when the relative FRET (%) was plotted as a function of Zn(II) concentration. The graph was fitted to one-site binding hyperbolic equation of Origrin (ver 9.0, OrugubLab Corporation): *y* = (FRET_max_·*x*)/(*K*_d_ + *x*), where *y* is FRET ratio and *x* is Zn(II) concentration.

### 2.3. Protein Kinase Assay

Prior to the protein kinase assay, a stock solution of PKA was diluted to 25 U·(unit)·µL^−1^. The protein kinase reaction was initiated by adding PKA (2 μL) to the peptide substrate (20 µM T-PEP1-Bio) dissolved in a reaction buffer (50 mM Tris-HCl, pH 7.5 containing 10 mM MgCl_2_ and 200 µM ATP). The reaction mixture was incubated at 30 °C for 90 min. Then, 100 µL of a 50% slurry streptavidin (SA) bead solution were initially washed with washing buffer (20 mM Tris-HCl, pH 7.4) twice and were added to the mixture and incubated for 30 min at RT to induce the biotin-avidin affinity reaction. After washing three times using centrifugation at 10,000 rpm for 2 min in order to eliminate MgCl_2_ and ATP, the peptide-conjugated SA beads were dispersed in 20 mM Tris-HCl buffer (pH 7.5) at an appropriate concentration. Afterward, QD525 (20 µL at 10 nM) and ZnCl_2_ (1 µL at 10 mM) were added to the solution containing peptide-conjugated SA beads to give a final volume of 100 µL, which was followed by 5-min incubation at RT and fluorescence measurement at wavelengths of 450–650 nm using a microplate reader (Varioskan^TM^ Flash, Thermo Scientific, Waltham, MA, USA).

### 2.4. Matrix-Assisted Laser Desorption/Ionization Mass Spectrometry (MALDI-MS) Analysis

A C18 pipette-tip was used to concentrate and desalt the peptide substrates with or without kinase reaction according to the manufacturer’s specifications. The C18 tip was wetted in 0.5% TFA/50% ACN and activated in 0.5% TFA/distilled water. Then, 10 µL of the sample reactant was adsorbed onto the C18 tip and rinsed in 0.5% TFA/50% ACN. Elution of the target peptide from the C18 tip was conducted directly onto a standard stainless steel MALDI target by dispensing about 0.7 µL of a matrix solution containing 1 mg of CHCA in 0.5% TFA/50% ACN. Mass spectrometric analysis of peptides was performed using an Axima-CFR (Shimadzu, Kyoto, Japan).

## 3. Results and Discussion

### 3.1. Detection Principle and Metal Affinity-Based QD-FRET

**Scheme 1:**
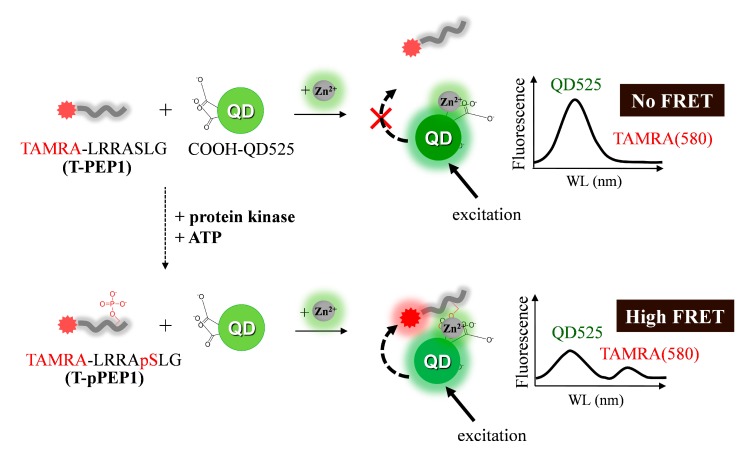
Schematic of Zn(II)-driven quantum dot-fluorescent resonance energy transfer (QD-FRET) after phosphorylation of peptide substrates. Once TAMRA-LRRASLG (T-PEP1) is phosphorylated by protein kinase on a serine residue, the resulting phosphopeptide causes a strong association with the surface groups (carboxyl groups) of QDs via Zn(II) coordination, leading to a high FRET signal between the QD and the TAMRA via the selective excitation of QDs.

**Figure 1:**
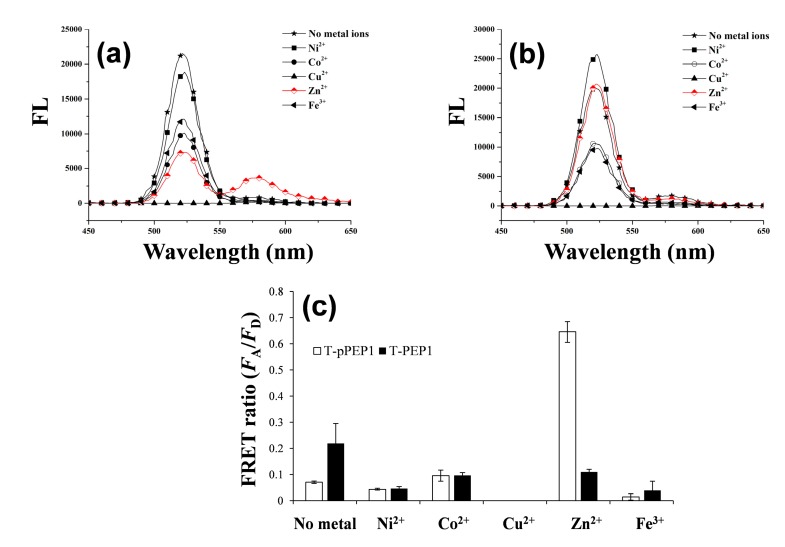
Fluorescent spectra of QDs with either (**a**) TAMRA-LRRApSLG (T-pPEP1); or (**b**) TAMRA-LRRASLG (T-PEP1) in the presence of different metal ions; (**c**) Effect of metal ions on the QD-FRET ratios of T-pPEP1 (white bar) and T-PEP1 (black bar). The FRET ratio was determined by the acceptor (*F*_A_) emission area (integrated from 550 to 650 nm) relative to the donor (*F*_D_) emission area (integrated from 450 to 550 nm). The concentrations of QD, T-pPEP (or T-PEP), and ZnCl_2_ were 2 nM, 80 nM and 100 μM, respectively. The QD-FRET spectra were obtained at an excitation wavelength of 380 nm.

**Figure 2:**
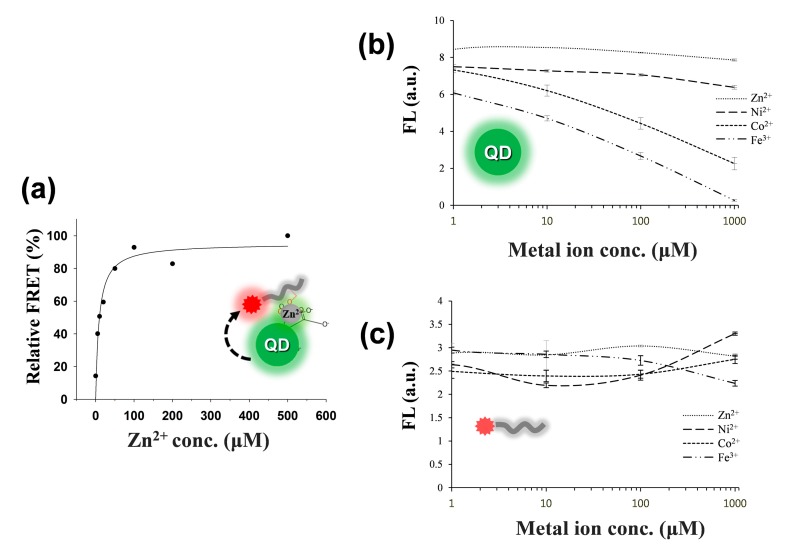
Changes in FRET (**a**) between QD and T-pPEP1 as a function of Zn^2+^ concentration. The relative FRET percentage was calculated by dividing the experimental FRET ratio by the maximal FRET ratio (0.74). Fluorescence intensities of donor (QD); (**b**) and acceptor (T-pPEP1); (**c**) as a function of metal ion (Zn^2+^, Ni^2+^, Co^2+^, and Fe^3+^) concentration. The concentrations of QD and T-pPEP1 were 2 nM and 80 nM, respectively. Excitation/emission wavelengths of QD-FRET (**a**); QD (**b**); and T-pPEP1 (**c**) were obtained at 380/580, 380/525, 530/580 nm, respectively.

As depicted in Scheme 1, once a dye-tethered peptide substrate is phosphorylated by a protein kinase, the addition of Zn(II) may lead to a strong FRET signal between the QD as an energy donor and the dye as an energy acceptor, whereas the unphosphorylated peptides may not cause the FRET. To explore this possibility, a synthetic peptide substrate labeled with 5(6)-carboxytetramethylrhodamine at the N-terminus (TAMRA-LRRASLG, termed T-PEP1) was compared with its phosphorylated form (TAMRA-LRRApSLG, termed T-pPEP1) (Figure 1). This peptide sequence, originating from porcine liver pyruvate kinase [26], was used as a substrate for protein kinase A (PKA). While divalent metal ions (Ni(II), Co(II), Cu(II), and Zn(II)) and a trivalent metal ion (Fe(III)) were tested, only Zn(II) ion triggered a strong association between the energy donor and acceptor of the QD-FRET in the presence of T-pPEP1 (Figure 1a and b). Since Cu^2+^ completely quenched the fluorescence intensity of QDs, there were no signals in QD-FRET at both acceptors. This Zn(II)-coordination led to a high FRET ratio (*F*_A_/*F*_D,_ 0.65), which is defined by the acceptor (*F*_A_) integrated emission relative to donor (*F*_D_) integrated emission (Figure 1c), whereas other metal ions did not produce a significant FRET ratio. In addition, non-phosphopeptides resulted in a marginal FRET ratio even in the presence of Zn(II). This result strongly indicates that Zn(II) is specifically associated with phosphopeptides on the surfaces of QDs. In the present study, the FRET ratio was saturated at a 1:40 molar ratio of QD to T-pPEP1 in the presence of Zn(II) ion when the concentration of QD was fixed to 2 nM (data not shown). The FRET ratio was also dependent on Zn(II) concentration, where maximum FRET ratio was acquired over the range of >100 µM Zn(II) at a 1:40 molar ratio of QD to T-pPEP1 (Figure 2a). Considering the hydrodynamic diameter (10–20 nm) and multivalent capacity of carboxyl QD525, 40 times more fluorescent peptides were not fully saturated relative to QDs, but this number was optimized between QD/T-pPEP1 and QD/T-PEP1 in terms of signal-to-background ratio. Compared to Zn(II)-caged complexes capable of capturing the phenyl phosphate dianion [27], free Zn(II) ions in this study revealed a relatively low binding affinity of *K*_d_ = 8.8 µM for T-pPEP1, based on the FRET efficiency. When we examined the effect of metal ions (except but Cu^2+^) on the florescence intensity of donor QD or acceptor dye, Zn^2+^ and Ni^2+^ ions did not affect the emission intensities of QD and T-pPEP1 (Figure 2b and c), whereas Co^2+^ and Fe^3+^ reduced the emission intensity of QD as their concentrations increased (Figure 2b). As a consequence, since free Zn(II) ions even at high concentration did not influence fluorescent intensity of the donor QD or the acceptor T-pPEP1, they are favorable for use in the FRET-based method in order to avoid synthesis of complex chemicals, which may adversely affect the fluorophores. Therefore, this result suggests that our Zn(II)-mediated QD-FRET would be useful for detecting phosphopeptides without complex metal-chelating ligands.

**Figure 3:**
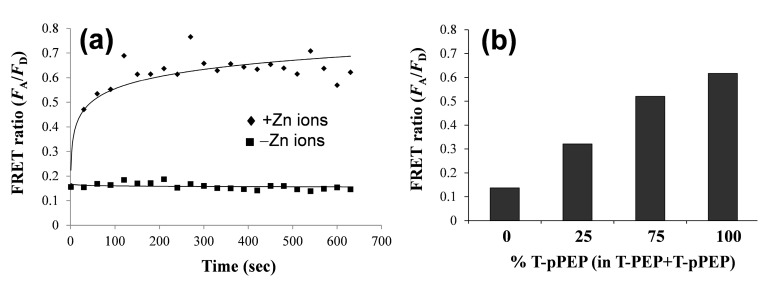
Time-dependent change in the QD-FRET ratio in the presence (black diamond) and absence (black square) of Zn(II) (**a**); and peptide phosphorylation-dependent change in the QD-FRET ratio (**b**). Total concentration of peptides (T-pPEP1 and T-PEP1) was kept constant at 80 nM, while T-pPEP1 concentration was varied (0%, 25%, 75%, and 100%). The concentrations of QD and metal ions were 2 nM and 100 μM, respectively. The QD-FRET spectra were obtained at an excitation wavelength of 380 nm.

**Figure 4:**
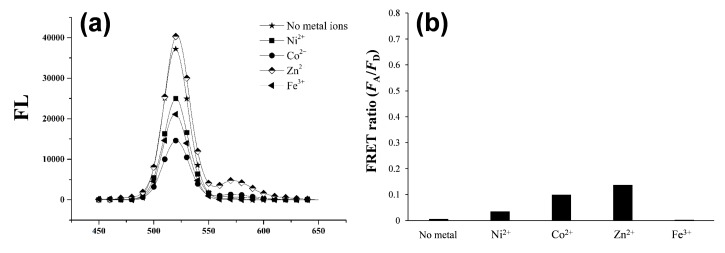
Fluorescent spectra (**a**) and corresponding QD-FRET ratio; (**b**) when carboxy QD525 was mixed with T-pPEP2 (TAMRA-IpYAAPKKG) in the presence of different metal ions. The concentrations of QD, T-pPEP2, and metal ions were 2 nM, 80 nM and 100 μM, respectively. The QD-FRET spectra were obtained at an excitation wavelength of 380 nm.

To examine kinetics and phosphorylation-dependency of this FRET phenomenon, we examined time-dependent FRET ratio in the presence and absence of Zn(II) ion (Figure 3a), and compared the FRET ratios at varied proportions of phosphorylated peptide (Figure 3b). The maximum FRET ratio was reached within 5 min after addition of Zn(II) ion, whereas no addition of Zn(II) did not change the FRET ratio. Under the same conditions, where the molar ratio of QD to peptide was 1:40 in the presence of Zn(II), the FRET ratio was proportional to the T-pPEP1 concentration. To gain insight into the detection of phosphorylation in other peptide substrates, another TAMRA-coupled peptide substrate (TAMRA-IYAAPKKG, termed T-PEP2) for Abl kinase and its phosphorylated form on its tyrosine residue (TAMRA-IpYAAPKKG, termed T-pPEP2) were also investigated under the same conditions of QD/Zn(II) (Figure 4). However, in contrast to that of T-PEP1/T-pPEP1, there was only a marginal difference in FRET ratio between T-PEP2 and T-pPEP2 in the presence of Zn(II). The low FRET efficiency in T-pPEP2 was not clearly understood in this study, but we assumed that QD-FRET efficiency via Zn(II) coordination would be susceptible to different phosphorylated amino acids.

### 3.2. Changes in QD-FRET Ratio by Phosphorylation Degree

**Figure 5:**
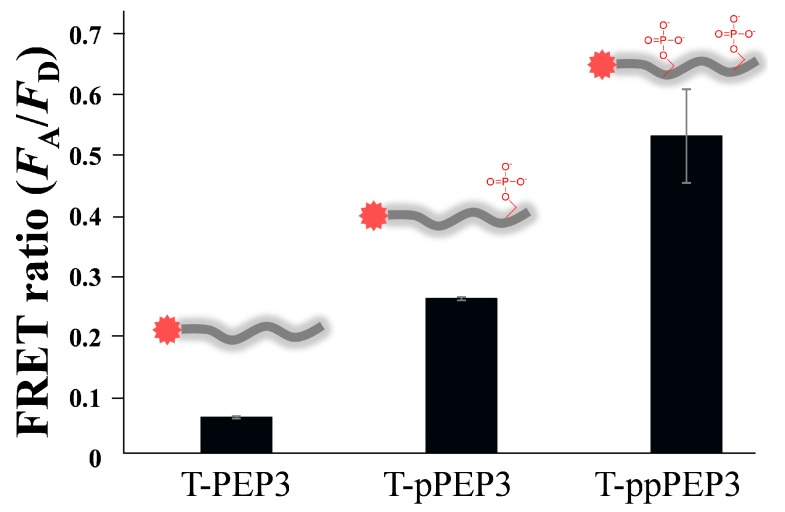
Comparison of FRET ratio between carboxyl QDs and three peptides via Zn(II)-coordination: TAMRA-KEEPPSPPQSPR (T-PEP3), TAMRA-KEEPPSPPQpSPR (T-pPEP3), and TAMRA-KEEPPpSPPQpSPR (T-ppPEP3). The concentrations of QD, peptide, and ZnCl_2_ were 2 nM, 80 nM and 100 μM, respectively. The QD-FRET signals were measured at an excitation wavelength of 380 nm.

To further examine the effect of phosphorylation on QD-FRET efficiency, a different serine-containing peptide substrate (TAMRA-KEEPPSPPQSPR, termed T-PEP3) and its two synthetic phosphorylated forms (TAMRA-KEEPPSPPQpSPR, *i.e.*, T-pPEP3 and TAMRA-KEEPPpSPPQpSPR, *i.e.*, T-ppPEP3) were compared based on FRET ratio in the presence of QD/Zn(II) (Figure 5). Since the annotated peptide sequence (KEEPPSPPQSPR) was derived from heat shock factor 1 protein that was constitutively controlled by two consecutive kinases (mitogen-activated protein kinase and glycogen synthase kinase 3) [28,29], the difference between the three peptides in terms of FRET ratio would provide insight into the relationship between the phosphorylation degree and FRET efficiency. Notably, the diphosphorylated peptide showed a relatively higher FRET ratio (0.53 ± 0.07) compared to those of mono-phosphorylated and unphosphorylated peptides, indicating that the Zn(II)-coordinated QD-FRET discriminated between the phosphorylation degrees of the same peptide substrate. With respect to the background FRET ratio (0.07 ± 0.002) of T-PEP3, its value was similar to that (0.10 in Figure 1c) of T-PEP1 in the presence of Zn(II). On the other hand, since the phosphorylation of this peptide started from a serine residue close to the C-terminus, the single phosphate ion of T-pPEP3 was distant from TAMRA dye compared to that of T-pPEP1. Intriguingly, this may explain why the FRET ratio (0.27) from T-pPEP3 was lower than that (0.65) of T-pPEP1 because the FRET efficiency, in general, is inversely proportional to the sixth power of the distance between donor and acceptor. The diphosphorylated peptide can also trigger the increased binding affinity via Zn(II)-phosphate complex, leading to a high FRET efficiency, but, unexpectedly, the QD-FRET efficiency (0.53) in T-ppPEP3 was slightly lower than that (0.65) of T-pPEP1 in Figure 1c in the presence of same serine residue. Indeed, taking into consideration the distance between the phosphorylated amino acid and the TAMRA, FRET ratios in QD/T-ppPEP3 and QD/T-pPEP1 likely reflect the distance-dependent behavior between the QD and the TAMRA, rather than Zn(II)-phosphate ion affinity. In fact, the phosphorylated serine (QD-binding site) in T-pPEP1 was closer to the TAMRA than that in T-pPEP3. Although many other factors (e.g., electrostatic interactions) might be involved in the different FRET ratios with different peptides substrate, this result suggests that QD-FRET via Zn(II) coordination is governed by the distance between the terminal dye and its close phosphorylated residue on the peptides.

### 3.3. Protein Kinase Assay by QD-FRET

**Figure 6:**
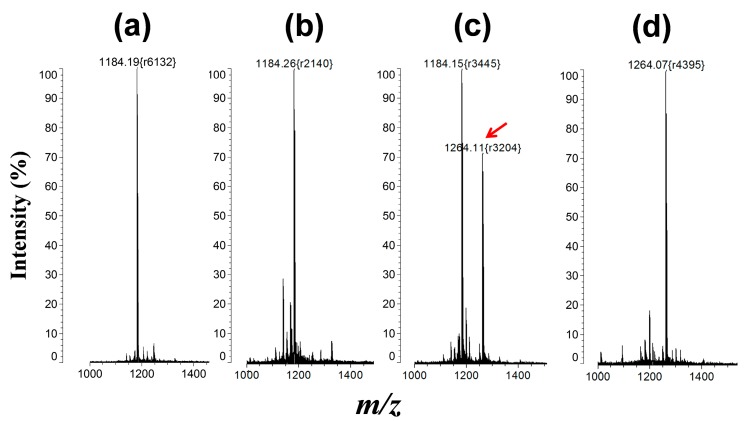
Phosphorylation of T-PEP1 (Mr = 1183) according to MALDI-MS analysis under different reaction conditions: (**a**) synthetic T-PEP1; (**b**) T-PEP1+PKA in the absence of ATP, (**c**) T-PEP1+PKA in the presence of ATP; and (**d**) synthetic T-pPEP1 (Mr = 1263). The phosphorylated molecular ion ([MH + HPO_3_]^+^) was observed at *m/z* 1264 in (**c**,**d**). The arrow indicates the phosphorylated peak. The r value in the round bracket means mass resolution.

**Figure 7:**
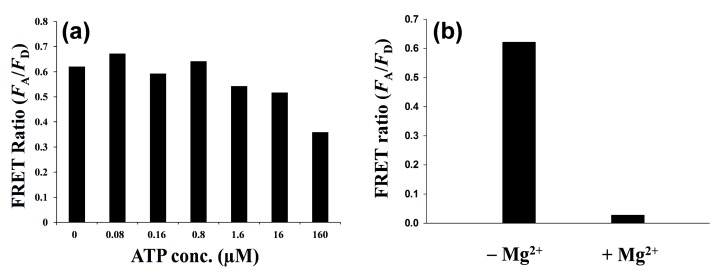
Effects of ATP (**a**) and Mg^2+^ (**b**) on the QD-FRET sensor signal output in the presence of T-pPEP1 and Zn(II). ATP with different concentrations or magnesium ion was added to the solution containing carboxyl QD525 (final 2 nM), T-pPEP1 (TAMRA-LRRApSLG, final 80 nM), and Zn^2+^ (final 100 µM) in the reaction buffer (20 mM Tris-HCl buffer, pH 7.4). The final concentration of Mg^2+^ was 10 mM.

The phosphorylation of T-PEP1 used in the presence of PKA was confirmed by matrix-assisted laser desorption/ionization mass spectrometry (Figure 6). Despite the phosphorylation of peptide substrate by protein kinase, detection of protein kinase activity using Zn(II)-specific QD-FRET was formidable because such Zn(II)-binding affinity was affected by other interferants in a reaction buffer, including competitive molecules (ATP or other phosphate derivatives), salts, surfactants, and non-metallic ions. In Figure 7a, as the ATP concentration increased to 160 µM in the reaction solution, which was prerequisite for a typical protein kinase reaction, the FRET ratio between QD and T-pPEP1 at a fixed concentration of Zn(II) decreased to 42% of the maximum ratio. In addition, non-metallic magnesium ions (Mg^2+^), which were essential for protein kinase activity, critically reduced the QD-FRET ratio (Figure 7b).

**Figure 8:**
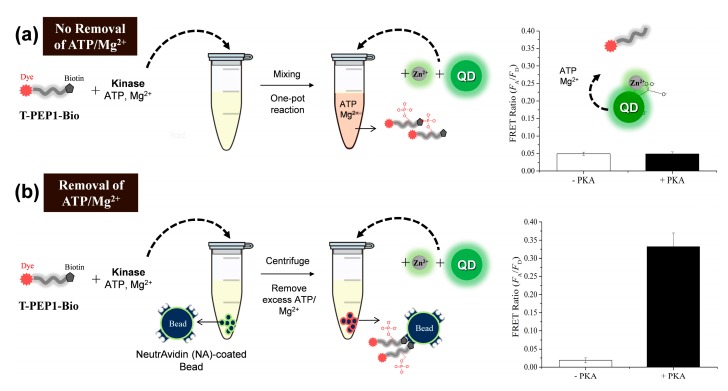
Bead-based protein kinase assay using QD-FRET with Zn(II): (**a**) one-pot protein kinase reaction without beads as a negative control and; (**b**) affinity-based protein kinase reaction using streptavidin (SA)-coated microbeads. A 20 µM peptide substrate (TAMRA-LRRASLGK-biotin, T-PEP1-Bio) was initially incubated in a reaction solution containing 0.5U µL^−1^ PKA, 10 mM MgCl_2_, 200 µM ATP in 20 mM Tris-HCl buffer (pH 7.4). The final concentrations of carboxyl QD and ZnCl_2_ were 2 nM and 100 μM, respectively. The QD-FRET signals were measured at an excitation wavelength of 380 nm. The error bars indicate the standard deviations in quadruplicate experiments.

To avoid these inherent problems, we employed an additional purification step using affinity-based peptide precipitation after a protein kinase reaction. As illustrated in Figure 8, T-PEP1 labeled with a biotin group (TAMRA-LRRASLGK-Biotin, termed T-PEP1-Bio) was subjected to a protein kinase reaction, followed by affinity-based purification of the reactant T-PEP1-Bio using streptavidin (SA)-coated microbeads. Consequently, the FRET ratio was measured by adding carboxyl QDs and Zn(II) to the peptide-attached SA-beads. In contrast to a one-pot reaction without SA-beads, where there were no FRET signals in the presence of PKA (Figure 8a), the use of SA-beads gave rise to a relatively high FRET under the same kinase condition (Figure 8b). This evidence indicates that SA-bead can be efficiently used to remove many adverse interferants through affinity precipitation and washing step. Thus, this bead-based method is suitable for the detection of peptide phosphorylation in a complex solution. However, in spite of the discernible FRET ratio by PKA activity, a relatively reduced FRET ratio (0.33), compared to that of synthetic phosphopeptide in Figure 1, may be attributed to less accessibility of QDs, which was caused by aggregation among the beads. Nonetheless, since the induced FRET ratio is higher than those of reported QD-FRET kinase assays [19,20], this strategy is favorable for assaying protein kinase activity.

In addition to the merits of QD-FRET, including ratiometric accuracy and high FRET efficiency, our approach enables a fast and simple detection of protein kinase activity without phosphate-specific antibodies. Most importantly, although many metal ions (or their complex forms) and metallic nanoparticles have been widely utilized in the enrichment of phosphopeptides by immobilized metal affinity chromatography and mass spectrometry [30,31], our method is preferable to the straightforward detection and identification of protein kinase activity. Moreover, the components used, consisting of free Zn(II) ions, commercially available carboxyl QDs, and synthetic short peptides, do not require complicated surface modifications and/or chemical synthesis, which otherwise limit general use. Given appropriate peptide substrates and further works, this method will have a potential for detecting other protein kinases and their inhibitors.

## 4. Conclusions

In conclusion, we demonstrated Zn(II)-mediated QD-FRET sensing of protein kinase activity. With neither complex chemical ligands nor surface modification of QDs, the phosphorylation of peptide substrates was easily detected by QD-FRET via Zn(II) coordination with high specificity, also leading to the prediction of phosphorylation degree in a single substrate. Furthermore, when the peptide substrate was combined with affinity-based purification, QD-FRET in the presence of Zn(II) enabled the rapid detection of protein kinase activity in an intermixed solution. Owing to its simplicity and general usability, our designed QD-FRET method is anticipated to facilitate applications for studying physiological functions of protein kinases in association with drug development.
